# Longterm Outcome of Therapeutic Vaccination with a Third Generation Pre-S/S HBV Vaccine (PreHevbrio^R^) of Chronically HBV Infected Patients

**DOI:** 10.3390/jpm14040364

**Published:** 2024-03-29

**Authors:** Hedwig Roggendorf, Daniel Shouval, Michael Roggendorf, Guido Gerken

**Affiliations:** 1 Institute of Molecular Immunology, University Hospital TUM, 81675 Munich, Germany; 2 Liver Unit, Hadassah Medical Center, POB 12000, Jerusalem 91120, Israel; shouval@hadassah.org.il; 3 Institute of Virology, Technical University of Munich/Helmholtz Zentrum, 81675 Munich, Germany; michael.roggendorf@tum.de; 4 Department of Gastroenterology, Helios Klinikum Niedernberg, 42551 Velbert, Germany; guido.gerken@helios-gesundheit.de

**Keywords:** chronic hepatitis B, NUC treatment, low-level HBsAg carriers, therapeutic vaccination, third generation pre-S/S vaccine, long-term observation

## Abstract

Several antiviral treatment regimens for chronic hepatitis B (CHB) virus infection have been shown to be effective in suppressing viral load and reducing the risk of hepatocellular injury and its complications. It has been hypothesized that high levels of circulating HBV surface antigen(s) may lead to immune tolerance against HBV and contribute to chronic carriership. Conversely, low-level HBsAg may create a window for the reconstitution of an HBV-specific immune response through vaccination and control of infection. Previous studies in non-responders to yeast-derived HBV vaccines, using a third-generation pre-S/S vaccine, have led to up to 95% anti-HBs seroconversion. This report evaluates the long-term outcome after experimental vaccination with a pre-S/S HBV vaccine intended as a therapeutic intervention in chronic HBV carriers. Four low-level HBsAg carriers (<500 IU/mL) were vaccinated three to seven times with 20 μg PreHevbrio^R^. Three out of four carriers eliminated HBsAg completely and seroconverted to anti-HBs. One patient seroconverted to anti-HBs but remained with a borderline HBsAg titer (10 IU/mL). Serum anti-HBs levels following repeated vaccination varied between 27 and >1000 IU/L, respectively. Long-term observation (>6 years) showed that after discontinuing NUC treatment for at least two years, HBsAg and HBV DNA remained negative with anti-HBs positive titers ranging between 80 and >1000 IU/L. Based on our preliminary observations, there is a rationale to further evaluate the role of this vaccine as a therapeutic agent.

## 1. Introduction and Rational

Currently, chronic HBV infection affects an estimated 300 million people worldwide, leading to about 1 million deaths per year caused by complications of liver disease and hepatocellular carcinoma (HCC) [1]. For more than 30 years, hepatitis B vaccines have efficiently decreased the incidence worldwide, also in countries known to be heavily affected (endemic) for HB, such as China [2]. Yet, a large pool of individuals with chronic infections remain at risk of developing liver cirrhosis and hepatocellular carcinoma. Those with chronic HBV infections serve as the main reservoir for viral spread. In the United States, fewer than one-third of individuals with chronic HBV are aware of their infection status, increasing the likelihood of transmission to susceptible populations and putting close family members, household contacts, and sexual partners at risk of infection [1].

An effective HBV-specific T-cell response is regarded as a key determinant in resolving acute infection. The human immune system has the potential to resolve an acute HBV infection in about 90% of infected individuals. However, in patients who fail to resolve acute infection and develop chronic hepatitis B, an effective T-cell response against the virus is functionally impaired or absent. Furthermore, a humoral anti-HBs immune response is required for protection against HBV infection due to exposure or re-exposure in patients who recover from acute hepatitis B, as well as in patients successfully immunized against HBV.

Current available therapies for chronic hepatitis B include the short-term administration of pegylated interferon-alfa (PEG-IFNα) or long-term nucleoside analogues (NUC) treatment. Fortunately, the current generation of NUCs, such as Entecavir (ETV) and Tenofovir (TDF), effectively suppress viral replication with a high barrier to resistance in the majority of patients [3]. However, NUCs cannot reconstitute immunological control and completely eliminate HBV in patients. The infection of woodchucks with woodchuck hepatitis virus (WHV) is an important preclinical model for chronic hepatitis B since it has a very similar pathogenesis as HBV infection in humans. This includes a high rate of chronic infection in early life, frequent integration in the genome of hepatocytes, cccDNA in the replication cycle of the virus and development of HCC. Studies in patients and woodchucks indicate a high frequency of HBV DNA or WHV DNA integration into the host genome during chronic infection, respectively [4]. Both integrated viral DNA and free episomal are important sources for the continuous production of large amounts of envelope proteins (HBsAg or WHsAg), which remain unaffected by NUC therapy [5]. HBsAg, present in high levels, seems to induce an immune inhibitory effect on both adaptive and innate immune functions [6,7]. The secretion of the small surface antigen, especially in the early phase of infection, has been suggested to interfere with the generation of an effective immune response against HBV through the induction of immune tolerance toward viral antigens [8]. 

This tolerance can promote an enhanced viral load and paradoxically reduce the intensity of hepatocellular injury [9].

There are rare cases of chronically HBV-infected patients who spontaneously overcome HBV-specific immune tolerance and clear HBsAg [10]. Additionally, HBsAg clearance rates have been reported in the years following the discontinuation of NUC therapy [11,12], with such cessation proposed as a strategy to induce enhanced immune control. The highest HBsAg seroclearance rate in this context reached 39%, 4–5 years after discontinuation of NUC therapy [13]. However, a recent study by Jeng et al. evaluating the cessation of NUC monotherapy in HBeAg-negative and HBeAg-positive patients determined the annual HBsAg seroclearance rate to be only 1.78% [14]. A low baseline HBsAg concentration was the only statistically significant correlate for HBsAg seroclearance, indicating that a high HBsAg level may be an important factor in maintaining immunotolerance in chronic hepatitis B carriers.

This immune tolerance may explain, at least in part, why attempts at therapeutic vaccination with conventional HBsAg vaccines have failed in several clinical trials [15]. In pre-clinical trials in the mouse model, it has been shown that vaccination of HBV transgenic mice with a high level of HBsAg are associated with a low level of B- and T-cell response to HBsAg or the core protein and do not reduce HBsAg or induce anti-HBs [16].

Nevertheless, low-level HBsAg in HBV carriers may create a window to reconstitute an HBV-specific immune response through so-called therapeutic vaccination in a compassionate access program setting. 

It was therefore hypothesized that patients under NUC therapy, who have a low HBsAg load, may be good candidates for an effective therapeutic intervention through vaccination against HBV.

### 1.1. Development of Vaccines for Protection against Hepatitis B Virus Infection

First-generation, plasma-derived hepatitis B vaccines containing HBsAg harvested from patients with chronic Hepatitis B were developed in the late 1970s. In the mid-1980s, second-generation recombinant DNA hepatitis B vaccines were constructed in yeast transfected with HBV-DNA sequences coding for the small hepatitis B virus surface protein (SHBs). These vaccines have gradually replaced the first-generation plasma-derived [17] vaccines and are currently used for universal vaccination of newborns and adults in more than 170 countries worldwide [18].

According to WHO guidelines, successful seroprotection against HBV infection after vaccination is defined by an anti-HBs titer of ≥ 10 IU/L (following immunization with at least three vaccine doses). The same threshold anti-HBs level applies to the definition of protection against hepatitis B after resolution of “wild-type” HBV infection. While the majority of vaccinees develop protection against HBV, 5–10% do not respond to the currently used conventional vaccines. These non-responders have anti-HBs levels < 10 IU/L after three or more injections with the conventional vaccine and remain susceptible to HBV infection. Thus, protection of such non-responders to conventional vaccination against HBV remains an important goal for specific risk groups (e.g., medical personnel involved in exposure-prone procedures, babies born to HBsAg positive mothers, or spouses of HBsAg carriers). The recently developed US FDA/EMA approved pre-S1/pre-S2/S HBV vaccine (PreHevbrio^R^) has been shown to bypass resistance to vaccination in such non-responders to conventional vaccines containing only the small surface antigen [17,19,20,21].

### 1.2. Development of Therapeutic Vaccines for Control of Chronic Hepatitis B

Therapy with NUCs does not completely eliminate HBV, and there is a need to explore additional therapeutic regimens. With a few exceptions, vaccines are traditionally used for prevention of infection and not for treatment of established infections. An early attempt at therapeutic vaccination with an HBV vaccine containing the small HBsAg was indeed unsuccessful [15], possibly due to a failure to generate an effective cellular immune response against HBV.

We hypothesized that a new third-generation highly immunogenic HBV vaccine, Sci-B-Vac™, recently renamed PreHevbrio^R^, containing all three envelope proteins, may harbor a therapeutic potential against HBV, possibly leading to a functional cure of HBV infection [20,21]. Indeed, restoring an antiviral B-cell and T-cell response not only in non-responders to the vaccine but also in patients with persistent HBV infection, remains a major challenge.

Consequently, starting in 2010, we initiated an experimental “Compassionate Access Program” in patients with chronic hepatitis B (CHB) with low-level HBsAg (<500 IU/mL) to assess the safety and efficacy of pre-S/S (PreHevbrio^R^) vaccination in chronic carriers [22]. This therapeutic vaccination was not structured as a clinical trial. 

Our early results reveal that a functional serologic cure was induced in a small number of HBsAg-positive patients treated with NUCs and immunized repeatedly with the pre-S1/pre-S2/S vaccine. We hypothesized that ongoing vaccinations using the pre-S/S vaccine in combination with NUC may lead to a functional cure of HBV. Consequently, we initiated a long-term follow-up of such patients.

The desired clinical outcome of our treatment protocol is the loss of HBsAg and induction of seroconversion to anti-HBs, leading to a putative “functional cure” as a result of suppression of viral replication with an aim to induce a sterilizing cure [23], eliminating hepatocytes containing episomal covalently closed circular HBV DNA (cccDNA).

## 2. Materials and Methods

### 2.1. Patients

An anonymous data request was sent to two virologic diagnostic laboratories to determine the frequency of low-level carriers of HBsAg who might be good candidates for this therapeutic vaccination protocol [22].

Four patients with chronic, low-level HBsAg were included in this pilot “Compassionate Access Program” using the third-generation pre-S/S HBV vaccine (PreHevbrio^R^). According to the Declaration of Helsinki § 37: “In the treatment of an individual patient, where proven interventions do not exist or other known interventions have been ineffective, the physician, after seeking expert advice, with informed consent from the patient or a legally authorised representative, may use an unproven intervention if in the physician’s judgement it offers hope of saving life, re-establishing health or alleviating suffering. This intervention should subsequently be made the object of research, designed to evaluate its safety and efficacy. In all cases, new information must be recorded and, where appropriate, made publicly available”. 

We followed this guideline in our clinical observation. All patients to be vaccinated with the pre-S/S vaccine (PreHevbrio^R^) gave written consent to this procedure. 

These patients were identified through the surveillance system of the registry at the University Hospital Essen and the Helios Klinikum Niedernberg. After screening our cohort of patients, only low-level carriers were chosen to give them this vaccine as compassionate use. There were no long-term HBsAg measurements prior to start of vaccination and HBV DNA below detection limit (HBV DNA “negative” as it is written in clinical diagnostic reports) was a prerequisite prior to vaccination. 

Among them, two were females and two were males, with ages ranging between 43 and 68 years. Baseline characteristics (age, gender, HBV-DNA, HbsAg, anti-HBs status, NUC therapy, e.g., Baraclude, Viread, Entecavir, Tenofovir) of the four vaccinated patients with low-level HBsAg before and after vaccination are shown in Table 1. All patients were HBeAg-negative and anti-HBc-positive.

The following inclusion criteria were used for recruitment: HBsAg-positive carrier status for more than five years; quantitative HBsAg level currently below 500 IU/mL; NUC treatment (e.g., Baraclude, Viread, Entecavir, Tenofovir) for at least two years; HBV DNA-negative; HBeAg-negative; anti-HBs-negative.

As controls, two high-level carriers of HBsAg (27,000 IU/mL and 8552 IU/mL, respectively) were also vaccinated. These patients were treated with NUCs (Entecavir) and had HBV-DNA below the detection limit. Baseline characteristics (age, gender, HBV-DNA, HBsAg, anti-HBs status, NUC therapy: Entecavir, Tenofovir) of these two vaccinated control patients (C 1, C 2) with high-level HBsAg before and after vaccination are provided in Table 2.

### 2.2. Pre-S1/Pre-S2/S HBV Vaccine 

The third-generation vaccine pre-S1/pre-S2/S HBV (PreHevbrio^R^) is an aluminum hydroxide adjuvanted recombinant hepatitis B vaccine [18], currently manufactured by VBI Vaccines Ltd., Cambridge, MA, USA. This pre-S1/pre-S2/S vaccine is produced in mammalian Chinese hamster ovary (CHO) cells transfected with appropriate HBV sequences that code for the three HBV envelope proteins: the small S hepatitis B surface antigen (SHBs), the middle pre-S2 (MHBs), and the large pre-S1 envelope protein (LHBs). The purified HBsAg particles, secreted by the transfected CHO cells, mainly consist of SHBs (75–77% p24, gp27), MHBs (17–21% gp33, gp36), and LHBs (3–7% p39, gp42).

This pre-S1/pre-S2/S vaccine was approved by the FDA in November 2021.The European Medicines Agency (EMA) authorized this vaccine for use in the EU (including Germany) since 18.5.2022. Authorization details: EMA Product Nr: EMEA/H/C/005466 Reference Number: EMA/129611/2022.

### 2.3. Vaccination Protocol and Monitoring 

**Figure 1:**
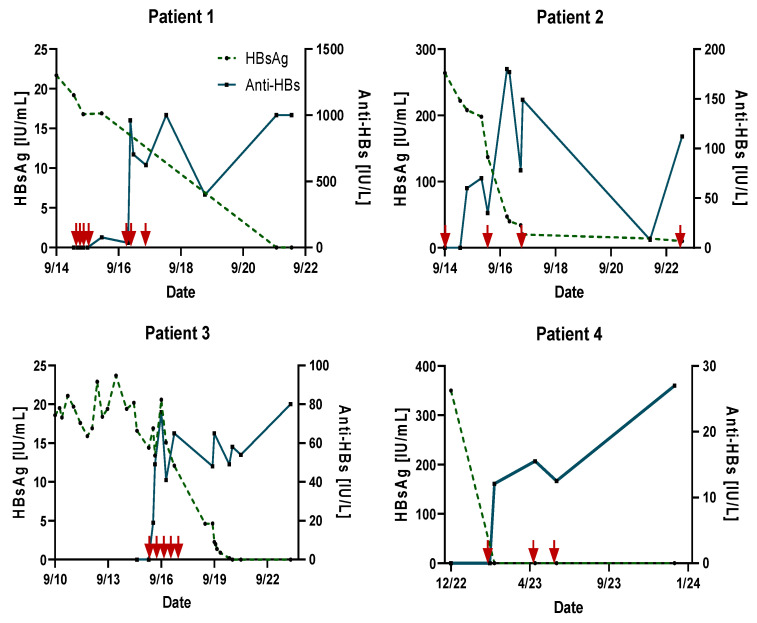
Kinetics of HBsAg and anti-HBs in vaccinated chronic low-level (HBsAg < 500 IU/mL) HBV carriers over time. Red arrows indicate the timing of vaccination/re-vaccination. The x-axis represents the month/year.

**Figure 2:**
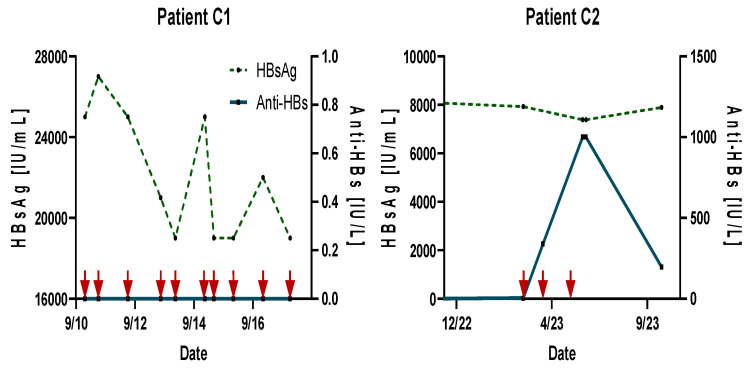
Kinetics of HBsAg and anti-HBs in vaccinated chronic high-level (HBsAg > 5000 IU/mL) HBV carriers over time. Red arrows represent time of vaccination/re-vaccination. The x-axis represents the month/year.

In this “Compassionate Access Program,” four patients who met the inclusion criteria were initially vaccinated according to the protocol on Day 0, Day 30, and Day 90. As there is no experience at which time intervals and how frequent therapeutic vaccination is needed to reach functional cure status, we used different schedules and numbers for vaccination. The numbers of vaccinations for each patient are given in Figure 1 and Figure 2. All patients were presented individually because they started therapy in different years. In Patient 1, we started with a single dose of 10 μg, but he showed no response in terms of anti-HBsAg. Therefore, he was vaccinated more frequently and consecutively with 20 μg. After that, we administered 20 μg of PreHevbrio^R^ initially in the other patients, which showed better response.

Anti-HBs levels (ECLIA) were determined 4 weeks after each vaccination. If a low immune response was observed after three vaccine doses (defined as an anti-HBs level < 100 IU/L), additional vaccine shots were administered. HBsAg (CMIA) and HBV DNA levels (CobasX800) were determined before vaccination. Monitoring of transaminases (GOT, GPT, GGT) and bilirubin was performed simultaneously (Table 3 and Table 4).

## 3. Results

Four patients, who were low-level carriers of HBsAg with levels less than 500 IU/mL (ranging from 18 IU/mL to 350 IU/mL), underwent vaccination with the PreHevbrio^R^ vaccine. They received between three and ten doses of 20 µg each, following pre-treatment with nucleos(t)ide analogues (NUCs). The inclusion criteria for these patients included: ongoing antiviral NUC therapy for more than 2 years; undetectable HBV-DNA; negative HBeAg; HBsAg levels under 500 IU/mL; and normal liver enzymes (GOT/GPT) below 50 U/L.

All four HBV carriers, with undetectable HBV DNA and under NUC treatment, who received three or more doses of the vaccine, seroconverted to anti-HBs at varying levels, as detailed in Table 1. The administration of the vaccine was well-tolerated, with no patients reporting serious adverse events (AEs), either locally or systemically, at any point during the study.

Patient 1, a 46-year-old male, was HBsAg-positive (22 IU/mL) prior to vaccination. After receiving seven doses over two years, he completely eliminated HBsAg and seroconverted to anti-HBs with levels greater than 1000 IU/L (see Figure 1). NUC treatment with Tenofovir was discontinued two years after the last vaccination. The total observation period for this patient was eight years.

Patient 2, a 68-year-old male, was HBsAg-positive (264 IU/mL) before vaccination. After three doses over 2.5 years, he seroconverted to anti-HBs (180 IU/L), and his HBsAg levels dropped to borderline reactive levels (10 IU/mL). His NUC treatment with Tenofovir was stopped. Over a monitoring period of six years, his anti-HBs level decreased to 8 IU/L. Following a booster shot, his anti-HBs levels increased to 112 IU/L two weeks after vaccination (see Figure 1). The total observation period for this patient was nine years.

Patient 3, a 43-year-old female, had an HBsAg level of 18 IU/mL before vaccination. After five doses over 1.5 years, she cleared HBsAg and seroconverted to anti-HBs with a level of 80 IU/L (see Figure 1). Her NUC treatment with Tenofovir was discontinued two years post-vaccination. The total observation period for this patient was eight years.

Patient 4, a female with an initial HBsAg level of 350 IU/mL, seroconverted to anti-HBs (27 IU/L) after three doses over four months and completely eliminated HBsAg (see Figure 1). She has been followed for one year so far, with ongoing NUC treatment with Entecavir. The total observation period for this patient has been one year.

### 3.1. Monitoring Unvaccinated Carriers

Given that a small number of NUC-treated patients may spontaneously clear HBsAg [24], we monitored four such unvaccinated low-level HBsAg carriers under NUC treatment for over two years. No significant change in HBsAg concentration was observed during this period.

### 3.2. Follow-Up of Vaccinated Patients with or without Ongoing NUC Treatment

Patients with chronic hepatitis B who discontinue treatment with NUCs often experience a relapse of HBV replication, characterized by the reappearance of HBV DNA and increased HBsAg levels in the serum [3]. Consequently, we monitored our vaccinated patients who discontinued NUC treatment for two to three years after their last vaccination.

Patient 1 remained HBsAg- and HBV DNA-negative six years after ceasing NUC treatment, with an anti-HBs titer of >1000 IU/L documented five years post-vaccination.

Patient 2 dropped his HBsAg level to a borderline level of 14 IU/mL one year post-vaccination and his anti-HBs Titer was 180 IU/L. NUC treatment was stopped two years after his last vaccination.

Patient 3 HBsAg level dropped to a borderline 14 IU/mL one year post-vaccination, with an anti-HBs titer of 180 IU/L. NUC treatment was halted two years after the final vaccination.

Patient 4 lost HBsAg but maintained low anti-HBs titers; thus, NUC treatment continues. Having recently completed the baseline vaccination series, further monitoring and potential re-vaccination are planned.

### 3.3. Vaccination of High-Level HBsAg Carrier Controls

Prior research has demonstrated that high concentrations of HBsAg can significantly suppress the immune response [24]. To verify ongoing immune tolerance during and after vaccination in patients with high levels of HBsAg (Table 2), we vaccinated a male chronic HBV carrier (Patient C1) with an extremely high HBsAg concentration of 27,000 IU/mL. Despite receiving ten doses of 20 µg PreHevbrio^R^ over seven years, there was no notable reduction in HBsAg nor seroconversion to anti-HBs (Figure 2).

Another patient (C2), a 65-year-old female initially HBsAg-positive (8552 IU/mL) before vaccination, maintained similar HBsAg levels (7896 IU/mL) after vaccination. This patient eventually seroconverted to anti-HBs > 1000 IU/L, which decreased to 197 IU/L within three months. Due to her high HBsAg levels, NUC treatment is ongoing.

## 4. Discussion

In this pilot observation, we followed four HBV carriers with low-level HBsAg (<500 IU/L) and undetectable HBV DNA, who were under NUC treatment, for up to 6 years after an attempt of therapeutic vaccination with the third-generation vaccine Pre-Hevbrio^R^. Remarkably, all four carriers seroconverted to anti-HBs, showing increasing antibody concentrations. This indicates a breakthrough in immune tolerance against HBsAg, which dropped below detection levels in patients 1, 3, and 4, while remaining at very low levels (10 IU/mL) in patient 2. The persistence of circulating HBsAg in patient 2 may be partly explained by the continuous secretion of HBsAg, potentially encoded by integrated HBV DNA—a phenomenon indistinguishable from HBsAg synthesized by episomal HBV-DNA.

The long-term observations of patients 1, 2, and 3 suggest the potential establishment of a functional cure of HBV infection over six years of follow-up. Patient 4, while HBsAg-negative, exhibited low anti-HBs levels and is scheduled for further booster shots and regular anti-HBs monitoring. Ongoing surveillance of these patients will continue to monitor for HBV reactivation or the development of liver cirrhosis or hepatocellular carcinoma (HCC). Should the anti-HBs titer drop below 100 IU/L, additional booster doses will be considered.

Patients C1 and C2, who had initially high HBsAg titers (27,000 IU/mL and 8552 IU/mL, respectively) and did not meet the inclusion criteria for the “Compassionate Access Program,” were vaccinated with up to ten doses of PreHevbrio. Unlike the low-level HBsAg carriers, both patients showed no HBsAg reduction or clearance. However, a substantial anti-HBs titer (>1000 IU/L) was observed in Control Patient C2 over two months, despite no change in the high HBsAg level.

These outcomes suggest that the low HBsAg concentration in some patients may have a less tolerizing effect on the immune response compared to high-level carriers [25].

The results of this pilot study indicate that low-HBsAg levels in HBV carriers may be a prerequisite for therapeutic intervention through immunization with a preS/S vaccine. To validate this hypothesis, a significantly larger cohort of HBV carrier candidates will be necessary. We already conducted a preliminary survey to assess the number of candidates for such a trial for therapeutic intervention through vaccination: HBsAg levels were tested in two cohorts of patients, using data obtained at the Technical University Hospital in Munich (*n* = 351) and the University Hospital Duisburg-Essen (*n* = 1131). We identified that 30% of patients had HBsAg levels below 500 IU/mL [22], making them potential candidates for a larger therapeutic vaccination trial with PreHevbrio combined with NUC treatment.

The cumulative experience from several studies highlights that NUC monotherapy significantly reduces HBV DNA but often fails to decrease HBsAg levels in chronic HBV (CHB) [3,14]. Thus, exploring new strategies for suppressing HBsAg production, such as treatment with iRNA (Interference RNA) compounds or nucleic acid polymers (NAPs), is warranted [26,27,28,29]. NAPs therapy has shown promise in two clinical studies, leading to a 2–7 log reduction of HBsAg concentration in 12 patients [30,31].

In addition to a B-cell response in patient 1 [22], who exhibited the highest antibody response, we observed an HBV-specific T-cell response. However, T-cell responses in the remaining patients were below the cutoff. Despite using highly sensitive assays, the HBV-specific T-cell numbers may have been too low for detection. Further research is needed to confirm that HBV-specific CD4 and CD8 T cells contribute to sterilizing immunity against HBV, enhanced by NUC treatment in combination with a pre-S/S HBV vaccine.

The methodology employed in the current compassionate access program naturally has its limitations, including the inability to evaluate the impact of the vaccination protocol on HBV-cccDNA. Another important point of discussion is the role immunosenescence regarding responsiveness to vaccinations in general but especially to HBV vaccines [32]. Nonetheless, our findings suggest that vaccination with a pre-S/S HBV vaccine may contribute not only to a better vaccine efficacy but even more important to the functional cure of persistent HBV infection and warrants evaluation in future clinical trials alongside new antiviral agents aimed at disrupting cccDNA. Finally, we sought to determine if therapeutic vaccination of HBV carriers with a pre-S/S vaccine could result in long-lasting elimination of HBsAg and seroconversion to anti-HBs even after ceasing nucleoside therapy. Three patients (1, 2, and 3) discontinued Entecavir or Tenofovir treatment after their last vaccine dose. Two to five years post-NUC therapy, three patients remained anti-HBs positive, indicating a sustained B cell response without NUC therapy. The anti-HBs antibodies induced through preS/S vaccination may appear due to overcome the immune tolerance and neutralize minor amounts of circulating HBV, preventing de novo infection of previously uninfected hepatocytes. Further studies in a larger cohort of vaccinated patients with persistent HBV are needed to confirm the induction of a functional cure through long-term monitoring of viral replication, anti-HBs levels, and evaluating a specific T cell response.

Short overview:

- The results of the present observation revealed an excellent anti-HBs seroconversion response in three out of four HBsAg carriers immunized repeatedly with a PreS/S vaccine.
- Furthermore, these data suggest that low-level HBV carriership seems to be a pre-requisite for successful therapeutic vaccination resulting in functional cure.
- No patient in this long-term observation (1 to 6 years) had a relapse or deterioration of the clinical status.
- These findings provide strong support for the development of a larger prospective study focused on low-level HBV carriers, utilizing a pre-S/S HBV vaccine in combination with NUC therapy.

## Figures and Tables

**Table 1:** Summary of therapeutic vaccination in four patients.

Patient	HBV-DNA Quant. IU/L (CobasX800)	HBs-Ag IU/mL (CMIA)	Anti-HBs IU/L (ECLIA)
Patient 1 Male, 46 y			
Prior to Vaccination	Negative	22	Negative
Number of Vaccinations: 4 with PreHevbrio^R^ (10 μg)			
Number of Vaccinations: 3 with PreHevbrio^R^ (20 μg)			
Post-Vaccination (48 months)	Negative	Negative	1000
Termination of NUC Treatment (24 Months after Vaccination): 2021			
Patient 2 Male, 68 y			
Prior to Vaccination	Negative	264	Negative
Number of Vaccinations: 4 with PreHevbrio^R^ (20 μg)			
Post-Vaccination (60 months)	Negative	10	112
Termination of NUC Treatment (30 Months after Vaccination): 2014			
Patient 3 Female, 43 y			
Prior to Vaccination	Negative	21	Negative
Number of Vaccinations: 5 with PreHevbrio^R^ (20 μg)			
Post-vaccination (48 months)	Negative	Negative	58
Termination of NUC Treatment (24 Months after Vaccination): 2020			
Patient 4 Female, 61 y			
Prior to Vaccination	Negative	350	Negative
Number of Vaccinations: 3 with PreHevbrio^R^ (20 μg)			
Post-Vaccination (7 months)	Negative	Negative	27
NUC Treatment: Ongoing			

**Table 2:** Summary of therapeutic vaccination in two control patients.

Patient	HBV-DNA Quant. IU/L (CobasX800)	HBs-Ag IU/mL (CMIA)	Anti-HBs IU/L (ECLIA)
Patient C1 Male, 68 y			
Prior to Vaccination	Negative	24.000	Negative
Number of Vaccinations: 10 with PreHevbrio^R^ (20 μg)			
Post-Vaccination (40 months)	negative	24.000	Negative
Termination of NUC Treatment (32 Months after Vaccination): 2019			
Patient C2 Female, 65 y			
Prior to Vaccination	Negative	8442	Negative
Number of Vaccinations: 3 with PreHevbrio^R^ (20 μg)			
Post-Vaccination (6 months)	Negative	7687	>1.000
NUC Treatment: Ongoing			

**Table 3:** Summary of transaminases development in four patients.

Patient	GOT/GPT IU/L 10–50	GGT IU/L < 66
Patient 1 Male, 46 y		
Prior to Vaccination	41/60	45
Post-Vaccination (48 months)	39/47	43
Termination of NUC Treatment (24 Months after Vaccination): 2021		
Patient 2 Male, 68 y		
Prior to Vaccination	27/38	24
Post-Vaccination (60 months)	23/28	13
Termination of NUC Treatment (30 Months after Vaccination): 2014		
Patient 3 Female, 43 y		
Prior to Vaccination	24/16	12
Post-Vaccination (48 months)	21/14	10
Termination of NUC Treatment (24 Months after Vaccination): 2020		
Patient 4 Female, 61 y		
Prior to Vaccination	18/20	18
Post-Vaccination (7 months)	25/27	22
NUC Treatment: Ongoing		

**Table 4:** Summary of transaminases development in two control patients.

Patient	GOT/GPT IU/L 10–50	GGT IU/L < 66
Patient C1 Male, 68 y		
Prior to Vaccination	24/18	34
Post-Vaccination (2 months)	28/22	56
Termination of NUC Treatment (32 Months after Vaccination): 2019		
Patient C2 Female, 65 y		
Prior to Vaccination	23/17	19
Post-Vaccination (1.5 months)	24/12	12
NUC Treatment: Ongoing		

## Data Availability

Data is unavailable due to privacy.
